# Corrosion damage and life prediction of concrete structure in the coking ammonium sulfate workshop of iron and steel industry

**DOI:** 10.1038/s41598-023-30015-1

**Published:** 2023-02-17

**Authors:** Yao Lv, Ditao Niu, Xiguang Liu, Mingqiang Lin, Yue-chen Li

**Affiliations:** 1 School of Civil Engineering, Xi’an University of Architecture and Technology, Xi’an, China; 2 State Key Laboratory of Green Building in Western China, Xi’an University of Architecture and Technology, Xi’an, China; 3 School of Civil Engineering and Architecture, University of Jinan, Jinan, China

**Keywords:** Structural materials, Chemical engineering, Civil engineering

## Abstract

Iron and steel plants emit a large amount of CO_2_ and SO_2_ in the production process, and the high concentrations of acid gases lead to serious corrosion damage of concrete structures. In this paper, the environmental characteristics and corrosion damage degree of concrete in a 7-year-old coking ammonium sulfate workshop were investigated, and the neutralization life prediction of the concrete structure was carried out. Besides, the corrosion products were analyzed through concrete neutralization simulation test. The average temperature and relative humidity in the workshop were 34.7 °C and 43.4%, and they were 1.40 times higher and 1.70 times less than those of the general atmospheric environment, respectively. Both the concentrations of CO_2_ and SO_2_ were significantly different in various sections of the workshop, and they were much higher than those of the general atmospheric environment. The appearance corrosion and compressive strength loss of concrete were more serious in the sections with high SO_2_ concentration, such as vulcanization bed section and crystallization tank section. The neutralization depth of concrete in the crystallization tank section was the largest, with an average value of 19.86 mm. The corrosion products gypsum and CaCO_3_ were obviously visible in the surface layer of concrete, while only CaCO_3_ could be observed at 5 mm. The prediction model of concrete neutralization depth was established, and the remaining neutralization service life in the warehouse, synthesis section (indoor), synthesis section (outdoor), vulcanization bed section, and crystallization tank section were 69.21 a, 52.01 a, 88.56 a, 29.62 a, and 7.84 a, respectively.

## Introduction

CO_2_ and SO_2_ diffuse into concrete and react with the cement hydration products. CO_2_ converts Ca(OH)_2_, calcium silicate hydrate (C–S–H) and calcium aluminate into CaCO_3_^1–4^. SO_2_ reacts with all calcium compounds of hydration products, including CaCO_3_, and converts them into sulfur-bearing compounds^5–7^. The list of sulfur-bearing compounds mainly includes calcium sulfite (CaSO_3_·1/2H_2_O), calcium sulfates (CaSO_4_, CaSO_4_·1/2H_2_O and CaSO_4_·2H_2_O) and calcium sulfoaluminates (3CaO·Al_2_O_3_·CaSO_4_ 12H_2_O and 3CaO·Al_2_O_3_·3CaSO_4_·31–32H_2_O)^7^.

Both carbonation and sulfuration of concrete can reduce the pore-solution pH^6–9^, and calcium sulfoaluminates are difficult to exist stably due to the decrease of pore-solution pH^10^. It was reported that the disappearance of ettringite (3CaO·Al_2_O_3_·3CaSO_4_·31–32H_2_O) and monosulfoaluminate hydrate (3CaO·Al_2_O_3_·CaSO_4_·12H_2_O) at 20 °C were at pH ≤ 10.7 and pH ≤ 11.6, respectively^11^. The pH ranges of ettringite that could stably exist at 25 °C, 50 °C, and 85 °C were 10.43–12.52, 10.52–12.41, and 10.87–12.25, respectively^12^. The decomposition of ettringite due to concrete carbonation was reported by authors^13–15^, and the reaction products were CaCO_3_, gypsum and alumina gel.

The pore-solution pH of concrete is usually in the range of 12.5–13.8^16–18^, where the thin protective film of iron oxides around rebar is stable. The decrease of pore-solution pH due to carbonation and sulfuration leads to the destabilization of passive film on the rebar. Once pH falls to about 9, the rebar begins to corrode due to the breakdown of passive film^19^. Therefore, it is necessary to development technologies and strategies for CO_2_ and SO_2_ resistance of concrete. To achieve this target, the experimental study and field investigation on the neutralization of concrete under the combined action of CO_2_ and SO_2_ should be carried out.

There are a few experimental studies on the neutralization of concrete under the combined action of CO_2_ and SO_2_, and they are exposed to an artificial atmosphere with the high concentrations of corrosion media^20,21^. The diffusion rate of CO_2_ in concrete was higher than that of SO_2_ under the combined action of CO_2_ and SO_2_. The main reason was that the CO_2_ concentration was much higher than that of SO_2_ in the industrial environment^7,20^. Meanwhile, the diffusion rate of CO_2_ with the same volume concentration was faster than that of SO_2_. Leah^21^ observed the gas diffusion process in concrete under the condition of CO_2_ and SO_2_ with the same volume concentration. It was found that concrete combined with CO_2_ at the initial stage of the reaction, and the carbonation product CaCO_3_ was generated continuously. Subsequently, SO_2_ converted CaCO_3_ into gypsum. Therefore, CO_2_ reacted with hydration products at first, and the essence of concrete sulfuration was the reaction between SO_2_ and carbonation product.

There is few field investigation on the concrete neutralization under the combined action of CO_2_ and SO_2_. Pavlik^7^ evaluated the degradation of concrete by flue gas from coal combustion in a power plant stack. It was observed that the concrete was divided into soft disintegrated zone, sulfated zone, carbonated zone, and un-neutralized zone. The pH, corrosion products, and microstructure in various zones were analyzed, respectively. Ren^22^ found that the concrete cover of underground stovepipe in a sintering plant was partially cracked or even peeled off, and the steel bars were seriously corroded.

In conclusion, the research results on the concrete neutralization under the combined action of CO_2_ and SO_2_ mainly focus on the neutralization mechanism, and the performance deterioration and life prediction of concrete should be carried out. The environmental characteristics of the local industrial environment should be monitored, and the concrete neutralization simulation test method should be proposed according to these environmental characteristics. Moreover, the corrosion damage degree, corrosion mechanism and life prediction of concrete under the action of CO_2_ and SO_2_ should also be accomplished.

In this paper, the environmental characteristics and corrosion damage of the concrete structures in a 7-year-old coking ammonium sulfate workshop of Wuhan Iron and Steel Corporation (WISCO) were investigated. The environmental characteristics such as temperature, relative humidity, CO_2_ concentration, and SO_2_ concentration were surveyed. Besides, the appearance, neutralization depth, and compressive strength of concrete were analyzed in various sections of the workshop. The concrete neutralization simulation test method was proposed according to the environmental characteristics, and the corrosion products were analyzed by using X-ray diffraction (XRD) and thermogravimetric analysis (TG). The prediction model of neutralization depth of concrete in the ammonium sulfate workshop was established, and the remaining neutralization service life in various sections was predicted.

## Experiments

### In situ test

#### Project profile

The iron and steel industry emits a large amount of CO_2_ and SO_2_ in the process of coking. Taking a 7-year-old coking ammonium sulfate workshop of WISCO as an example, the environmental characteristics and corrosion damage of concrete were investigated from August to October 2013. WISCO was located in Qingshan District, Wuhan City. The ammonium sulfate workshop was built in 2006, and the structural style was a three-layers concrete frame structure.

**Figure 1:**
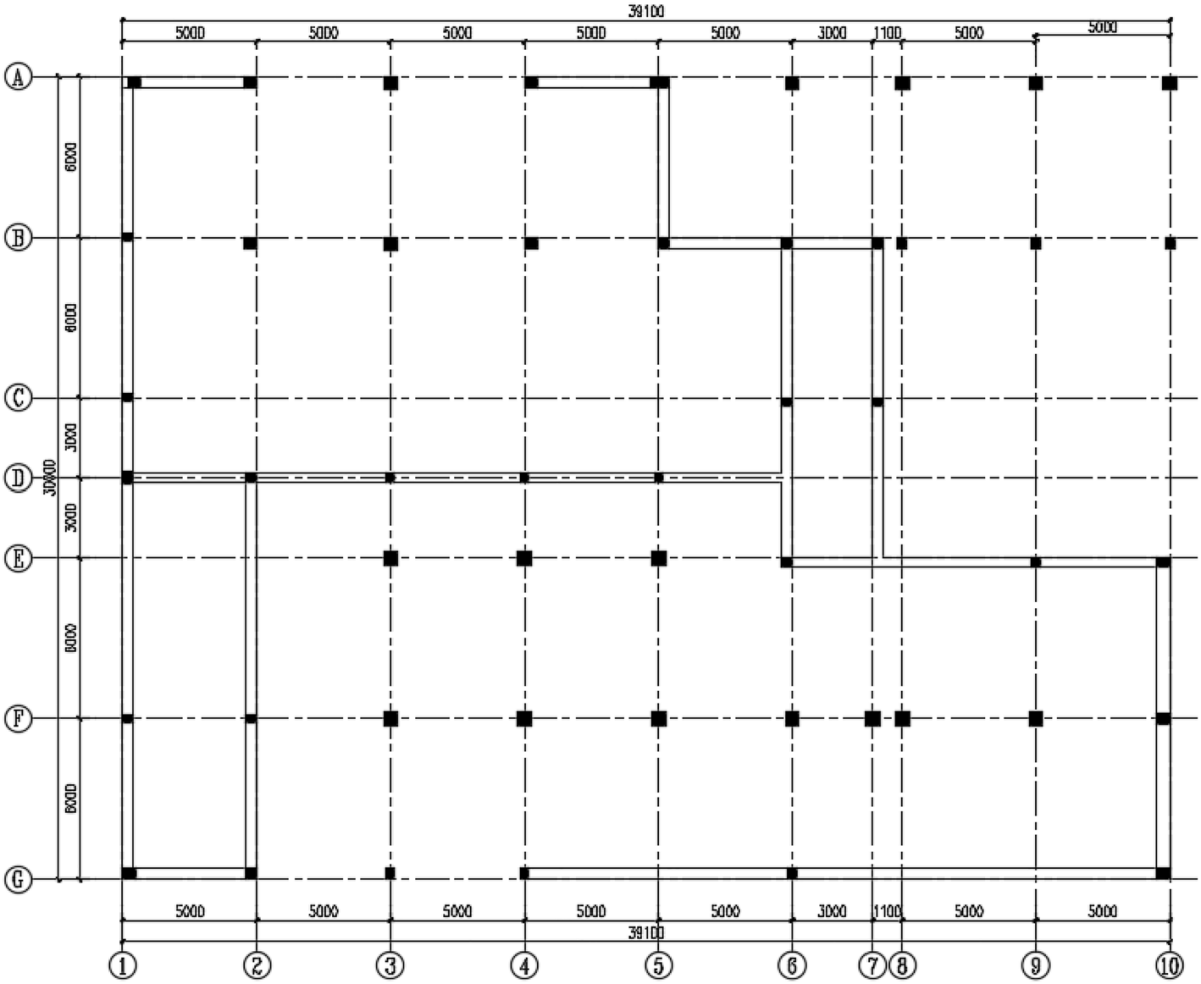
The structure plan of the ammonium sulfate workshop.

The structure plan is shown in Fig. 1. As shown in the figure, the length and width of the workshop were 39.1 m and 30 m, respectively. The process layout of the workshop was as follows: The A–D axes of the first floor were the ammonium sulfate synthetic section, and the synthesis section within the 7–10 axes was in an open air environment. The D–G axes of the first floor were the warehouse. The second floor of the workshop was the vulcanization bed section, and the third floor was the crystallization tank section. The serial number of the concrete column is MN-R. M and N are the axes numbers (M = A, B,…, G; N = 1, 2,…, 10), and R is the floor numbers (R = 1, 2, 3).

#### Materials

The concrete consisted of P.O. 42.5 ordinary Portland cement, with natural river sand as the fine aggregate and crushed limestone as the coarse aggregate. The pumping agent was used with a water reducing rate of 18%. The mixture proportion of concrete is listed in Table 1. The thickness of concrete cover was 30 mm. The strength grade of concrete was C30, and the characteristic value of compressive strength of concrete was 30 MPa.

**Table 1:** Mixture proportion of concrete.

W/C	Cement/(kg/m^3^)	Fly ash/(kg/m^3^)	Fine aggregate/(kg/m^3^)	Coarse aggregate/(kg/m^3^)	Water/(kg/m^3^)	Pumping agent/(kg/m^3^)
0.41	300	100	780	1077	163	8.30

#### Testing methods

##### Temperature and relative humidity monitoring

The temperature and relative humidity in the ammonium sulfate workshop were tested by using a temperature and humidity recorder, and the data were collected hourly. The layout of measuring points was based on Chinese standard GB/T 18204.13-2000^23^ and GB/T 18204.14-2000^24^. There were five measuring points in each section, and they were arranged in two diagonals with the shape of plum blossom. The height of measuring points was 0.8–1.6 m from the ground, and the distance between the measuring points and the heat source or wall was not less than 0.5 m. The average values of all measuring points were the average temperature and average relative humidity in the ammonium sulfate workshop, respectively.

##### Concentrations of CO_2_ and SO_2_ monitoring

The concentrations of CO_2_ and SO_2_ in various sections of the ammonium sulfate workshop were monitored by using a portable carbon dioxide detector and a portable sulfur dioxide detector, respectively. The layout of measuring points was the same as that for temperature and relative humidity monitoring, and the average values of all measuring points in various sections were the average CO_2_ concentration and average SO_2_ concentration, respectively.

##### Neutralization depth of concrete

The neutralization depth test method of concrete was adopted according to Chinese standard JCJ/T 23-2011^25^. Concrete columns were randomly selected to test neutralization depth, and each column had 3–7 testing zones. Take a hole with a diameter of 15 mm in each testing zone, and the depth of the hole should be larger than its neutralization depth. The neutralization depth was measured with a 1% phenolphthalein alcohol solution (alcohol solution contains 20% distilled water). Use a carbonation depth meter for measurement. Each hole was measured three times, and the average of three measurements was considered as the neutralization depth of the testing zone. The average of all testing zones for each column was the neutralization depth of concrete column.

##### Compressive strength of concrete

The compressive strength of concrete was tested by the rebound method according to JCJ/T 23-2011^25^. The selection of concrete column was the same as that of column for testing the neutralization depth. Ten testing zones were selected for each column, and they were arranged on two symmetrical measurable surfaces of the column. The measuring points in each testing zone were not less than 16. The measuring points should be evenly distributed, and the distance between two adjacent measuring points should not be less than 20 mm. The distance between the measuring points and the exposed steel bars or embedded parts was not less than 30 mm. The measuring points should not appear on stones and pores, and each measuring point only bounced once. Ignoring three maximum values and three minimum values, the mean value of the remaining measuring points was the average rebound value of the testing zone. The average rebound value of ten testing zones was the strength of the concrete column. The equivalent value of compressive strength of concrete was converted according to the rebound value of compressive strength and the neutralization depth.

### Simulation test

In order to analyze the corrosion mechanism of concrete in the ammonium sulfate workshop, the corrosion products of concrete under the combined action of CO_2_ and SO_2_ were studied by concrete neutralization simulation test. The raw materials and mixture proportion of concrete in the simulation test were the same as those in the ammonium sulfate workshop. The concrete neutralization simulation test method was proposed according to the concrete carbonation test method in Chinese standard GB/T 50082-2009^26^.

#### Testing parameters

The environmental parameters in the simulation test were set according to the actual environment of the ammonium sulfate workshop. The settings of the temperature and relative humidity in the simulation test were the same as the actual temperature and relative humidity in the workshop. Therefore, the temperature and relative humidity in the simulation test were 35 °C and 45%, respectively. The CO_2_ concentration in the simulation test was 20% according to GB/T 50082-2009^26^. The concentration of CO_2_ was 649 times higher than that of SO_2_ in the ammonium sulfate workshop. Therefore, the SO_2_ concentration in the simulation test was 0.03%. According to concrete carbonation test method in GB/T 50082-2009^26^, 4 days of the simulation test is equivalent to 7 years of the actual environment. Therefore, the test period was 4 days.

#### Testing procedure

The test procedure included the following steps:

- The cube specimens were dried at 60 °C for 48 h before the test.
- Each specimen was covered (except its two opposite sides) with epoxy resin to ensure that the diffusion of CO_2_ and SO_2_ in concrete was one-dimensional.
- The specimens were placed in the concrete carbonation test chamber, and the distance between adjacent specimens was at least 50 mm.
- The environmental parameters in the concrete carbonation test chamber were set as follows: temperature = 35 °C, relative humidity = 45%, and CO_2_ concentration = 20%.
- The specimens were taken out after 4 days.
- The carbonated specimens were placed in the concrete sulfuration test chamber.
- The environmental parameters in the concrete sulfuration test chamber were set as follows: temperature = 35 °C, relative humidity = 45%, and SO_2_ concentration = 0.03%.
- The test period of sulfuration test was 4 days.

#### Testing methods

##### XRD

The pulverizer was used to extract concrete powder. The specimen was powdered in different layers (1 mm/layer). The powder was passed through a 0.16 mm sieve. The powder was dried at 50 °C for 24 h before the test. The prepared powder was used in the XRD and TG test. XRD analysis was used to examine the phase composition of concrete. The diffraction used a Cu-Kα source, and the diffraction angle was from 5° to 45° at a step of 0.02°.

##### TG

TG method was used to measure the composition of concrete. The test was carried out at a temperature range from 30 to 900 °C, and the heating rate was 10 °C/min. The experimental atmosphere was nitrogen.

## Results and discussion

### Survey of environmental characteristics

**Figure 2:**
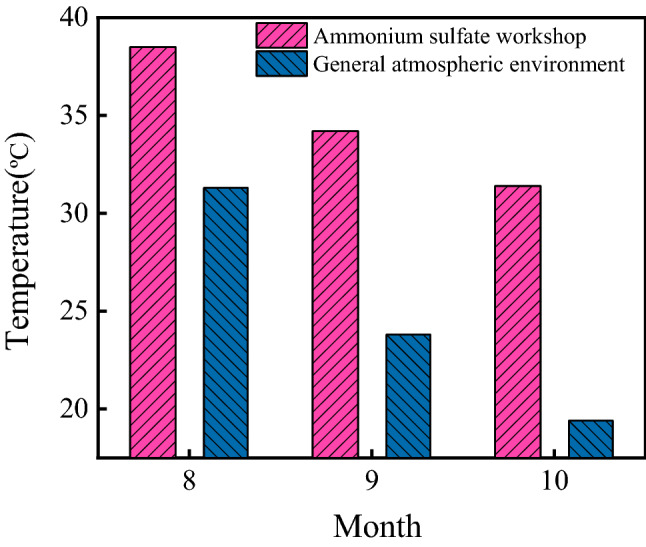
Temperatures in the ammonium sulfate workshop and general atmospheric environment.

Both the temperatures in the ammonium sulfate workshop and general atmospheric environment are shown in Fig. 2. As shown in the figure, the temperatures in the workshop in August, September, and October were 38.5 °C, 34.2 °C and 31.4 °C, respectively. During the same period, the atmospheric temperatures in Wuhan were 31.1 °C, 23.8 °C and 19.4 °C, respectively. The temperature in the workshop increased with the increase in that of the general atmospheric environment. The average temperature in the workshop was 34.7 °C, and it was 1.40 times higher than that of the general atmospheric environment.

**Figure 3:**
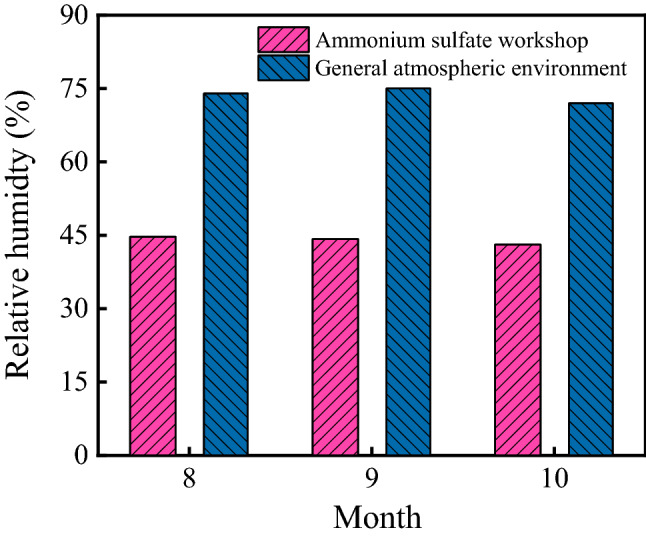
Relative humidity in the ammonium sulfate workshop and general atmospheric environment.

Both the relative humidity in the ammonium sulfate workshop and general atmospheric environment are shown in Fig. 3. As shown in the figure, the relative humidity in the workshop in August, September, and October were 44.7%, 44.2%, and 41.3%, respectively. During the same period, the relative humidity of the general atmospheric environment in Wuhan were 74%, 75%, and 72%, respectively. Both the relative humidity in the workshop and general atmospheric environment barely changed. The average relative humidity in the workshop was 43.4%, and the relative humidity of the general atmospheric environment was 1.70 times higher than that in the workshop.

**Figure 4:**
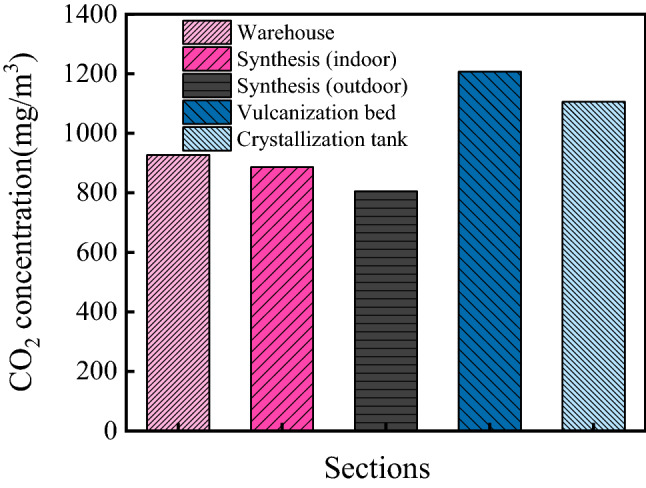
CO_2_ concentration in the ammonium sulfate workshop.

The CO_2_ concentrations in the ammonium sulfate workshop are shown in Fig. 4. As shown in the figure, the average CO_2_ concentration in the workshop was 986.2 mg/m^3^. Besides, the CO_2_ concentrations in various sections were significantly different, and the CO_2_ concentrations in the warehouse, synthesis section (indoor), synthesis section (outdoor), vulcanization bed section, and crystallization tank section were 927 mg/m^3^, 886 mg/m^3^, 805 mg/m^3^, 1207 mg/m^3^, and 1106 mg/m^3^, respectively. According to the relevant information from the Wuhan Environmental Protection Bureau (WHEPB), the average CO_2_ concentration of the atmospheric environment in Qingshan District was 740.5 mg/m^3^. Therefore, the CO_2_ concentration in the ammonium sulfate workshop was larger, and the maximum value was 1.49 times higher than that of the general atmospheric environment.

**Figure 5:**
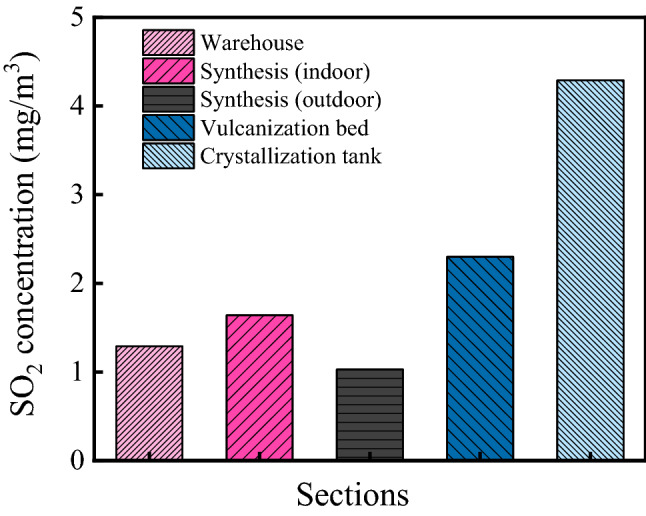
SO_2_ concentration in the ammonium sulfate workshop.

The SO_2_ concentrations in the ammonium sulfate workshop are shown in Fig. 5. As shown in the figure, the average SO_2_ concentration in the workshop was 2.11 mg/m^3^. There were significant differences in SO_2_ concentrations in various sections, and the SO_2_ concentrations in the warehouse, synthesis section (indoor), synthesis section (outdoor), vulcanization bed section, and crystallization tank section were 1.29 mg/m^3^, 1.64 mg/m^3^, 1.03 mg/m^3^, 2.30 mg/m^3^ and 4.29 mg/m^3^, respectively. According to the relevant information from the WHEPB, the average SO_2_ concentration of the atmospheric environment in Qingshan District was 0.056 mg/m^3^. Therefore, the SO_2_ concentration in the ammonium sulfate workshop was higher, and the maximum value was 76.6 times higher than that of the general atmospheric environment. In addition, the SO_2_ concentration was much lower than CO_2_ concentration in the ammonium sulfate workshop.

### Investigation on corrosion degree of concrete structure

#### Appearance of concrete

**Figure 6:**
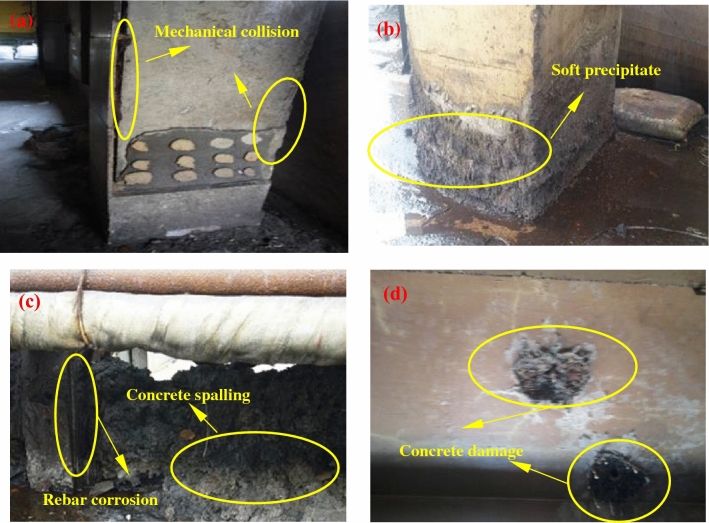
Appearance of concrete structure in the ammonium sulfate workshop. (**a**) The column in the warehouse; (**b**) the column in the synthesis section (outdoor); (**c**) the platform cornice in the vulcanization bed section; (**d**) the floor in the crystallization tank section.

The appearance of concrete in the ammonium sulfate workshop is shown in Fig. 6. As shown in the figure, there were only some damage on the concrete surface caused by mechanical collision in the warehouse (Fig. 6a). There were obvious expansion, spalling, and pulverization on the surface of concrete column in the synthesis section (outdoor), and a large amount of white crystals were precipitated (Fig. 6b). The platform cornice concrete in the vulcanization bed section was completely corroded and peeled off, and the steel bars were exposed and partially rusted (Fig. 6c). Although the floor in the crystallization tank section was coated, the coating was partially damaged, and the damaged concrete was severely corroded (Fig. 6d).

CO_2_ reacted with hydration products of concrete to form CaCO_3_, which made the pore structure denser than that of uncarbonated concrete. Therefore, carbonation could not change the appearance of concrete. However, SO_2_ converted CaCO_3_ into gypsum, which rapidly increased the solid-phase volume of concrete. A large amount of gypsum produced excessive internal stress in concrete, resulting in expansion and cracking on the concrete surface^6^. Therefore, the degree of appearance change of concrete increased as the SO_2_ concentration increased.

The SO_2_ concentration in the outdoor synthesis section was not high, but the outdoor environment was affected by rainwater. Water played a key role in the corrosion reaction. Water provided a medium for ion transport, and it was a necessary condition for reaction between SO_2_ and CaCO_3_. Moreover, the reaction products calcium sulfates were the substances containing crystal water. Calcium sulfates containing more crystal water increased the solid-phase volume of concrete, and concrete was more likely to crack. Relevant research showed that SO_2_ could cause serious corrosion damage of concrete when there was liquid water on the concrete surface^21^. Therefore, the appearance change in the outdoor synthesis section was relatively large.

#### Neutralization depth of concrete

**Figure 7:**
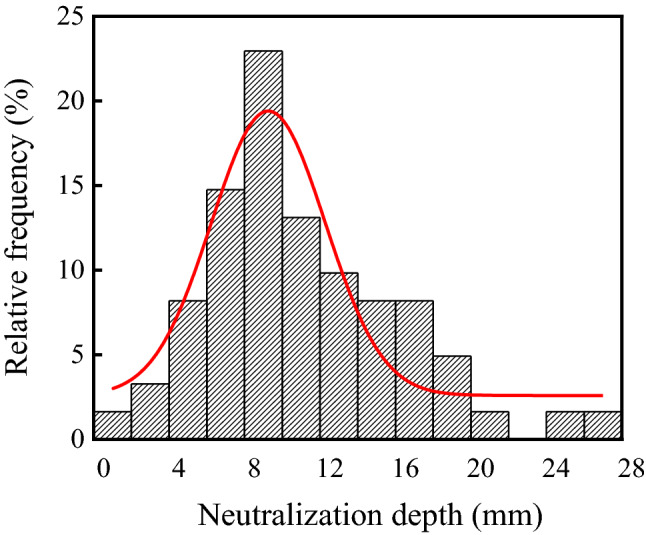
Frequency distribution histogram of concrete neutralization depth in the ammonium sulfate workshop.

The neutralization depth of concrete in the ammonium sulfate workshop is listed in Table 2, and the frequency distribution histogram of concrete neutralization depth is shown in Fig. 7. As shown in the table and figure, the average, standard deviation, and coefficient of variation of concrete neutralization depth in the workshop were 11.17 mm, 5.23 mm, and 0.47, respectively. Figure 7 showed that the p value was 0.09 at a significant level α = 0.05 by using Shapiro–Wilk. Therefore, the neutralization depth of concrete in the ammonium sulfate workshop followed the normal distribution.

**Table 2:** Neutralization depth of concrete in the ammonium sulfate workshop.

Sections	Serial number	Testing zones						
		1	2	3	4	5	6	7
Warehouse	F3-1	7.76	11.63	7.91				
	F4-1	0.93	11.59	7.60				
	F5-1	8.56	16.64	9.60				
	F6-1	13.60	15.03	8.57				
	F7-1	11.62	9.65	4.93				
	G3-1	9.69	5.65	6.94				
Synthesis section (indoor)	A3-1	6.77	8.25	14.33				
	A6-1	12.77	10.11	6.33				
Synthesis section (outdoor)	A7-1	5.11	11.47	13.42				
	A9-1	6.36	8.02	2.05				
	A10-1	9.52	9.01	11.62				
	B10-1	9.71	5.51	6.94	14.26	13.05	4.17	8.46
Vulcanization bed section	A2-2	17.33	11.25	9.79	12.05	3.14	19.22	7.90
	A3-2	14.15	11.53	9.33	14.85	16.91	12.40	17.62
Crystallization tank section	A2-3	24.22	16.90	19.51	21.87	9.93	26.89	19.72

**Figure 8:**
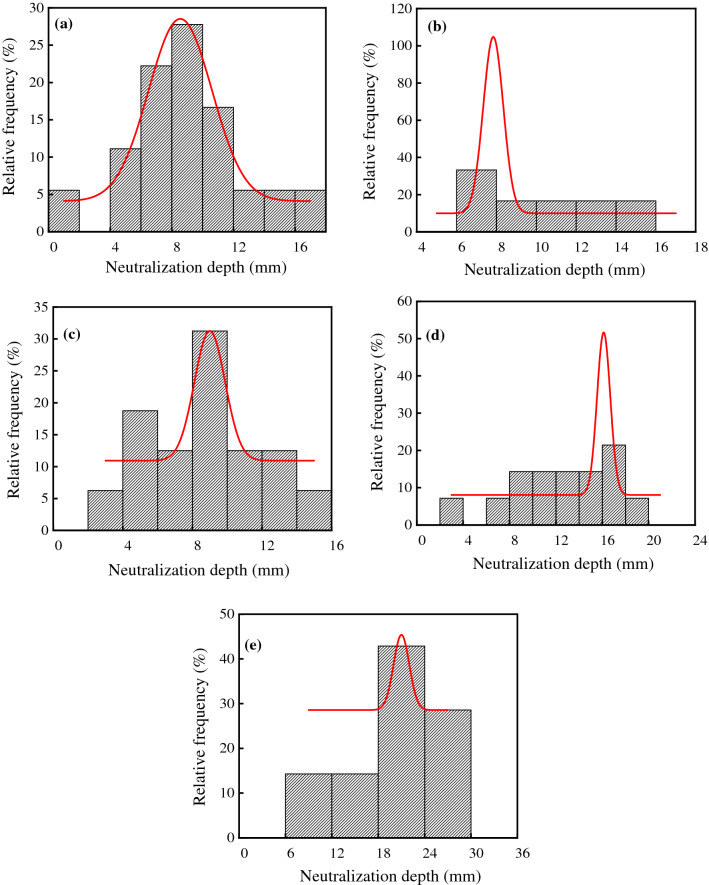
Frequency distribution histograms of concrete neutralization depth in various sections of the ammonium sulfate workshop. (**a**) Warehouse; (**b**) synthesis section (indoor); (**c**) synthesis section (outdoor); (**d**) vulcanization bed section; (**e**) crystallization tank section.

The frequency distribution histograms of concrete neutralization depth in various sections of the workshop are shown in Fig. 8. As shown in Table 2 and Fig. 8, the neutralization depths of concrete in the warehouse, synthesis section (indoor), synthesis section (outdoor), vulcanization bed section, and crystallization tank section were significantly different, and the average values were 9.33 mm, 9.76 mm, 8.67 mm, 12.68 mm and 19.86 mm, respectively. This was because the neutralization depth was related to the concentrations of acid gases. The CO_2_ concentrations in the vulcanization bed section and crystallization tank section were relatively high, and the neutralization depths were larger than those in the warehouse and synthesis section. In addition, the SO_2_ concentration in the crystallization tank section was higher than that in the vulcanization bed section, and the damage of concrete was more serious. CO_2_ diffused from mic-cracks into concrete, and the neutralization rate increased. Therefore, the neutralization depth of concrete in the crystallization tank section was 1.57 times larger than that in the vulcanization bed section.

As shown in Fig. 8, the p values in the warehouse, synthesis section (indoor), synthesis section (outdoor), vulcanization bed section, and crystallization tank section were 0.93, 0.52, 0.94, 0.82 and 0.79 at a significant level α = 0.05 by using Shapiro–Wilk. Therefore, the neutralization depth of concrete in various sections followed the normal distribution.

#### Compressive strength of concrete

**Figure 9:**
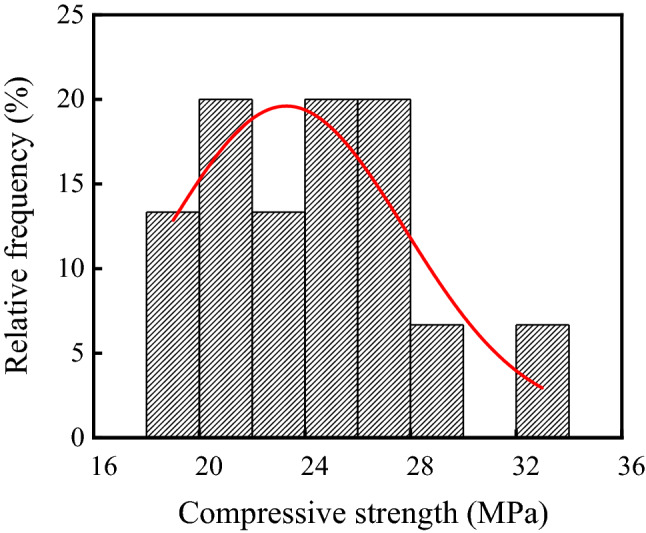
Frequency distribution histogram of concrete compressive strength in the ammonium sulfate workshop.

Compressive strength of concrete in the ammonium sulfate workshop is shown in Table 3, and the frequency distribution histogram of concrete compressive strength is shown in Fig. 9. As shown in the table and figure, the average and standard deviation of the compressive strength were 24.30 MPa and 3.74 MPa, respectively, and the coefficient of variation was 0.15. Figure 9 showed that the p value was 0.32 at a significant level α = 0.05 by using Shapiro–Wilk. Therefore, the compressive strength of concrete in the ammonium sulfate workshop followed the normal distribution.

**Table 3:** Compressive strength of concrete in the ammonium sulfate workshop.

Sections	Serial number	Rebound value/MPa	Neutralization depth/mm	Equivalent value/MPa
Warehouse	F3-1	40.0	9.10	25.0
	F4-1	40.0	6.71	25.0
	F5-1	43.0	11.60	28.9
	F6-1	37.0	12.40	21.3
	F7-1	35.0	8.73	19.2
	G3-1	39.0	7.43	23.7
Synthesis section (indoor)	A3-1	41.0	9.78	26.2
	A6-1	46.0	9.74	33.1
Synthesis section (outdoor)	A7-1	41.0	10.00	26.2
	A9-1	34.0	5.48	19.5
	A10-1	39.0	10.05	23.7
	B10-1	40.0	8.87	25.0
Vulcanization bed section	A2-2	41.0	11.53	26.2
	A3-2	37.0	13.83	21.3
Crystallization tank section	A2-3	36.0	19.86	20.2

As shown in Table 3, the compressive strength of concrete in various sections of the workshop decreased. Relevant studies showed that the carbonation of concrete increased the compressive strength^27,28^, while concrete sulfuration decreased the strength^29^. Therefore, the compressive strength of concrete in the crystallization tank section and vulcanization bed section was low due to the high concentration of SO_2_. Although the SO_2_ concentration in the synthesis section (outdoor) was the lowest, the strength dropped greatly due to the influence of rainwater, with an average value of 23.60 MPa.

### Analysis of corrosion products of concrete

#### XRD analysis

**Figure 10:**
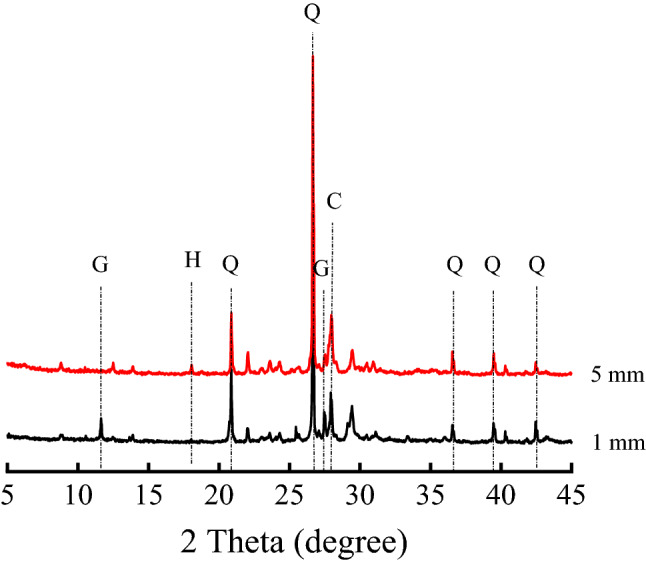
XRD patterns of concrete under the action of CO_2_ and SO_2_. *Q* Quartz (SiO_2_), *G* Gypsum (CaSO_4_ 2H_2_O), *C* Calcite (CaCO_3_), *H* Portlandite (Ca(OH)_2_).

The XRD patterns of concrete with different depths are shown in Fig. 10. As shown in the figure, the crystalline phases were gypsum (CaSO_4_·2H_2_O), calcite (CaCO_3_), portlandite (Ca(OH)_2_) and quartz (SiO_2_). Among them, Ca(OH)_2_ was the hydration product, and gypsum and CaCO_3_ were the corrosion products of concrete under the action of CO_2_ and SO_2_. The diffraction peaks of gypsum and CaCO_3_ were obviously visible in the surface layer of concrete, while the Ca(OH)_2_ diffraction peak was not obvious. The gypsum diffraction peak could not be observed at the depth of 5 mm, and the Ca(OH)_2_ peak appeared.

The CO_2_ concentration in the workshop was much higher than that of SO_2_, and therefore, the diffusion rate of CO_2_ in concrete was faster than that of SO_2_. CO_2_ reacted with the hydration product Ca(OH)_2_ to form CaCO_3_, and SO_2_ reacted with the carbonation product CaCO_3_ to form gypsum. Only CO_2_ could diffuse to a depth of 5 mm from the concrete surface, so CaCO_3_ was observed in the interior concrete. The concentrations of CO_2_ and SO_2_ in the surface layer were higher than those in the interior concrete, so Ca(OH)_2_ could not exist in the surface layer.

It might be well known that gypsum reacted with aluminate phase to form ettringite in concrete under sulfate attack^30–32^, while ettringite could not be observed in concrete under the action of CO_2_ and SO_2_. This was because CO_2_ and SO_2_ were dissolved in the pore solution and ionized into H^+^, which reacted with alkaline hydration products. Because of the decrease of pore-solution pH, ettringite could not exist stably, and it was decomposed into gypsum and an aluminum-containing gel^6,30^. Therefore, the sulfated product of concrete was only gypsum.

#### TG analysis

**Figure 11:**
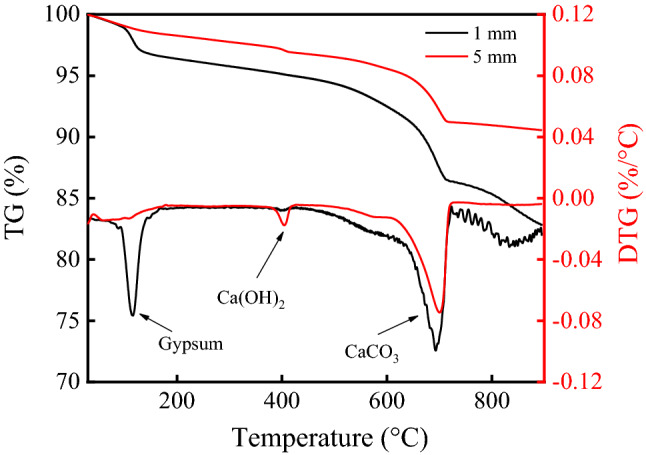
Thermal analysis curves of concrete under the action of CO_2_ and SO_2_.

The TG analysis results of concrete with different depths are shown in Fig. 11. Three main endothermic peaks were apparent in the TG and DTG curves, and they corresponded to gypsum, Ca(OH)_2_ and CaCO_3_, respectively. Among them, CaCO_3_ and gypsum were the corrosion products of concrete under the action of CO_2_ and SO_2_. The height of CaCO_3_ endothermic peak in the surface layer of concrete was higher than that at the depth of 5 mm. This might be due to the maximum CO_2_ concentration was in the surface layer of concrete. Therefore, although some CaCO_3_ in the surface layer of concrete reacted with SO_2_ to form gypsum, the content of CaCO_3_ was still larger than that at 5 mm.

### Life prediction of concrete structure

#### Prediction model of neutralization depth of concrete

A large number of researchers have verified that the carbonation depth of concrete is proportional to the square root of exposure time^1,33–36^. The carbonation depth of concrete is affected by temperature, relative humidity, CO_2_ concentration, and compressive strength of concrete in this experiment. According to the literature^36^, the carbonation depth of concrete in the ammonium sulfate workshop can be expressed as:

1 $$X_{{\text{C}}} = k_{{{\text{mc}}}} k_{{\text{e}}} k_{{{\text{CO}}_{{2}} }} k_{{\text{f}}} \sqrt t ,$$

where *X*_C_ is the carbonation depth of concrete, mm; *k*_mc_ is the calculation mode parameter, *k*_mc_ = 0.996; *k*_e_, *k*_CO2_ and *k*_f_ are the carbonation influence coefficients of temperature and humidity, CO_2_ concentration and compressive strength, respectively; *t* is the carbonation exposure time, d.

The carbonation influence coefficients of temperature and humidity, CO_2_ concentration, and compressive strength can be calculated in Eqs. (2)–(4), respectively^36^.

2 $$k_{{\text{e}}} = 2.56\sqrt[4]{T}RH\left( {1 - RH} \right),$$

3 $$k_{{{\text{CO}}_{{2}} }} = \sqrt {\frac{{C_{{\text{C}}} }}{0.03}} ,$$

4 $$k_{{\text{f}}} = \frac{57.94}{{f_{{{\text{cuk}}}} }} - 0.76,$$

where *T* is the temperature, °C; *RH* is the relative humidity, %; *C*_C_ is the concentration of CO_2_, %; and *f*_cuk_ is the compressive strength of concrete, MPa.

Substituting Eqs. (2)–(4) into Eq. (1), the carbonation depth of concrete in the ammonium sulfate workshop is given in Eq. (5).

5 $$X_{{\text{C}}} = 14.72\sqrt[4]{T}\left( {1 - RH} \right)RH\sqrt {C_{{\text{C}}} } \left( {\frac{57.94}{{f_{{{\text{cuk}}}} }} - 0.76} \right)\sqrt t .$$

Substituting the temperature, relative humidity, CO_2_ concentration, concrete compressive strength, and exposure time into Eq. (5), the carbonation depth of concrete in the ammonium sulfate workshop is calculated.

SO_2_ concentration in the ammonium sulfate workshop is much lower than that of CO_2_. However, the concrete neutralization rate is accelerated due to the expansion and cracking of concrete under SO_2_ attack. Therefore, the neutralization depth of concrete in the ammonium sulfate workshop can be expressed as:

6 $$X = K_{{\text{S}}} \sqrt {C_{{\text{S}}} } X_{{\text{C}}} ,$$

where *X* is the neutralization depth of concrete, mm; *K*_S_ is the neutralization influence coefficient of SO_2_; *C*_S_ is the concentration of SO_2_, %.

**Figure 12:**
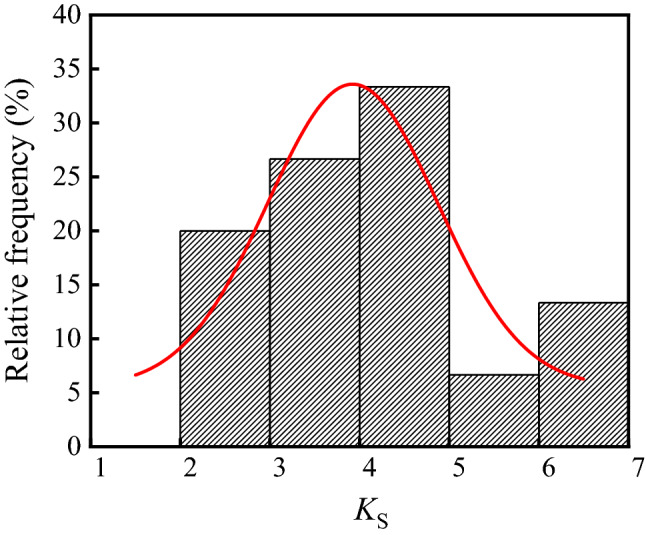
Frequency distribution histogram of *K*s.

Substituting the carbonation depths, neutralization depths, and SO_2_ concentration into Eq. (6), *K*_S_ is calculated and the frequency distribution histogram of *K*_S_ is shown in Fig. 12. Figure showed that the average, standard deviation, and coefficient of variation of *K*_S_ were 4.10, 1.31, and 0.32, respectively. Using Shapiro–Wilk method, the p value of *K*_S_ was 0.65 at a significant level α = 0.05. Therefore, *K*_S_ in the ammonium sulfate workshop followed the normal distribution.

In conclusion, the neutralization depth of concrete in the ammonium sulfate workshop is expressed as:

7 $$X = 14.72K_{{\text{S}}} \sqrt[4]{T}\left( {1 - RH} \right)RH\sqrt {C_{{\text{C}}} } \sqrt {C_{{\text{S}}} } \left( {\frac{57.94}{{f_{{{\text{cuk}}}} }} - 0.76} \right)\sqrt t .$$

#### Neutralization life prediction of concrete structure

The high alkalinity inside the concrete forms a passivation film for the rebar to protect it from being corroded. CO_2_ and SO_2_ dissolve in the pore solution and ionize into H^+^, which reacts with the OH^-^ ionized from Ca(OH)_2_, and neutralization reaction occurs in concrete. Once the concrete cover is completely neutralized, passivation film of rebar is destroyed, and therefore, the rebar begins to corrode. The end of the neutralization service life of concrete is the initial time of steel bar corrosion. Therefore, the limit state function of the neutralization service life of concrete is shown in Eq. (8).

8 $$c - x_{0} - X\left( t \right) = 0,$$

where, *c* is the thickness of concrete cover, mm; *x*_0_ is the neutralization remain, mm; *X*(*t*) is the neutralization depth of concrete, mm; and *t* is the exposure time, a.

According to the literature^36^, the neutralization remain of concrete is shown in Eq. (9).

9 $$x_{0} = 4.86\left( { - RH^{2} + 1.5RH - 0.45} \right)\left( {c - 5} \right)\left( {\ln f_{{{\text{cuk}}}} - 2.3} \right).$$

**Figure 13:**
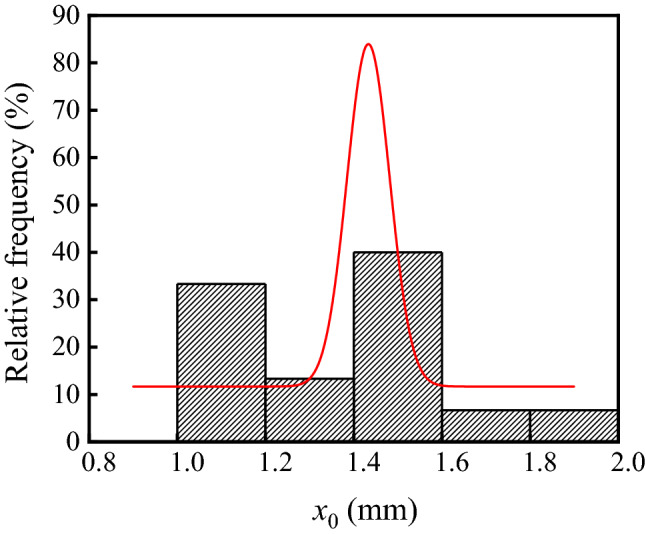
Frequency distribution histogram of *x*_0_.

Neutralization remains of concrete in the ammonium sulfate workshop are calculated, and the frequency distribution histogram of *x*_0_ is shown in Fig. 13. Figure showed that the average, standard deviation, and coefficient of variation of *x*_0_ were 1.35 mm, 0.23 mm, and 0.17, respectively. Using Shapiro–Wilk method, the p value of *x*_0_ was 0.54 at a significant level α = 0.05. Therefore, neutralization remains of concrete in the ammonium sulfate workshop followed the normal distribution.

Substituting the calculation results of Eqs. (7) and (9) into Eq. (8), the concrete neutralization service life in the warehouse, synthesis section (indoor), synthesis section (outdoor), vulcanization bed section, and crystallization tank section of the ammonium sulfate workshop were 76.21 a, 59.01 a, 95.56 a, 36.62 a, and 14.84 a, respectively. Therefore, the remaining neutralization service life in the warehouse, synthesis section (indoor), synthesis section (outdoor), vulcanization bed section, and crystallization tank section were 69.21 a, 52.01 a, 88.56 a, 29.62 a, and 7.84 a, respectively.

## Conclusions

In this study, the environmental characteristics and corrosion degree of concrete in a 7-year-old coking ammonium sulfate workshop were analyzed, and the neutralization life prediction of the concrete structure was carried out. Besides, the corrosion products of concrete under the action of CO_2_ and SO_2_ were studied. The main conclusions are as follows:

1. The temperature in the workshop increased with the increase in that of the general atmospheric environment. The average temperature in the workshop was 34.7 °C, and it was 1.40 times higher than that of the general atmospheric environment. Both the relative humidity in the workshop and general atmospheric environment barely changed. The average relative humidity in the workshop was 43.4%, and the relative humidity of the general atmospheric environment was 1.70 times higher than that in the workshop.
2. The average CO_2_ concentration and SO_2_ concentration in the workshop were 986.2 mg/m^3^ and 2.11 mg/m^3^, and they were 1.31 times and 37.68 times higher than those of the general atmospheric environment, respectively. In addition, both the concentrations of CO_2_ and SO_2_ in various sections of the workshop were significantly different.
3. The appearance of concrete in various sections of the workshop was significantly different. There were only some damage on the concrete surface caused by mechanical collision in the warehouse, and a large amount of white crystals were precipitated on the concrete surface in the synthesis section (outdoor). Platform cornice concrete was completely corroded and peeled off in the vulcanization bed section. The coating of the floor in the crystallization tank section was partially damaged, and the damaged part was severely corroded.
4. The neutralization depth of concrete in various sections of the workshop was significantly different and followed the normal distribution. The neutralization depth of concrete increased with the increase in the concentrations of CO_2_ and SO_2_, and the neutralization depth in the crystallization tank section was the largest, with an average value of 19.86 mm.
5. The compressive strength of concrete in various sections of the workshop decreased. The compressive strength of concrete in the crystallization tank section and vulcanization bed section was low due to the high concentration of SO_2_. The strength dropped greatly in the synthesis section (outdoor) due to the influence of the rainwater.
6. Gypsum and CaCO_3_ were the corrosion products of concrete under the action of CO_2_ and SO_2_. Gypsum and CaCO_3_ were obviously visible in the surface layer of concrete, while only CaCO_3_ could be observed at the depth of 5 mm. The content of CaCO_3_ in the surface layer of concrete was larger than that at the depth of 5 mm.
7. The prediction model of the neutralization depth of concrete was established, and the remaining neutralization service life of concrete in the warehouse, synthesis section (indoor), synthesis section (outdoor), vulcanization bed section, and crystallization tank section were 69.21 a, 52.01 a, 88.56 a, 29.62 a, and 7.84 a, respectively.

## Data Availability

The datasets used and analyzed during the current study available from the corresponding author on reasonable request.
